# MicroRNAs in Woody Plants

**DOI:** 10.3389/fpls.2021.686831

**Published:** 2021-08-31

**Authors:** Lisha Fang, Yanmei Wang

**Affiliations:** ^1^College of Forestry, Henan Agricultural University, Zhengzhou, China; ^2^Department of Biological Sciences, University of Wisconsin-Milwaukee, Milwaukee, WI, United States

**Keywords:** MicroRNAs, functions, woody plants, growth and development, abiotic and biotic stresses

## Abstract

MicroRNAs (miRNAs) are small (∼21-nucleotides) non-coding RNAs found in plant and animals. MiRNAs function as critical post-transcriptional regulators of gene expression by binding to complementary sequences in their target mRNAs, leading to mRNA destabilization and translational inhibition. Plant miRNAs have some distinct characteristics compared to their animal counterparts, including greater evolutionary conservation and unique miRNA processing methods. The lifecycle of a plant begins with embryogenesis and progresses through seed germination, vegetative growth, reproductive growth, flowering and fruiting, and finally senescence and death. MiRNAs participate in the transformation of plant growth and development and directly monitor progression of these processes and the expression of certain morphological characteristics by regulating transcription factor genes involved in cell growth and differentiation. In woody plants, a large and rapidly increasing number of miRNAs have been identified, but their biological functions are largely unknown. In this review, we summarize the progress of miRNA research in woody plants to date. In particular, we discuss the potential roles of these miRNAs in growth, development, and biotic and abiotic stresses responses in woody plants.

## Introduction

MiRNAs are small (∼21-nucleotides) non-coding RNAs that have emerged as key post-transcriptional regulators of gene expression in both plants and animals (Jones-Rhoades et al., 2006; Chen, 2008; Filipowicz et al., 2008; Ramachandran and Chen, 2008). In plants, a primary miRNA (pri-miRNA) is transcribed from a *MIRNA* (*MIR*) gene, forming a stem-loop structure. RNase III-type endonucleases (also known as Dicer proteins) release the stem segment to produce a paired precursor miRNA (pre-miRNA) of approximately 21 nucleotides and 2-nucleotide 3′overhangs. One strand of miRNA in the miRNA duplex becomes the mature miRNA, which is loaded into an RNA-induced silencing complex (RISC). The mature miRNA recognizes its target mRNA(s) by base pairing, resulting in its cleavage or translational attenuation. Some miRNAs also function in chromatin modification by mediating DNA methylation (Wu et al., 2010; Jia et al., 2011). A study of target transcripts of plant miRNAs found that most encode transcription factors involved in plant development patterns or cell differentiation (Rhoades et al., 2002). To date, a vast number of miRNAs have been identified from a variety of plants, including woody plants.

MiRNAs play remarkably widespread roles in controlling almost all aspects of plant growth and development, ranging from determining organ polarity to meristem function, vascular and root development, flower formation, seed and fruit development, and biotic and abiotic stress responses. In general, the functions of miRNAs are characterized by analyzing mutants with impaired miRNA biogenesis (Griffiths-Jones, 2004; Griffiths-Jones et al., 2006; Griffiths-Jones et al., 2008; Liu et al., 2010; Kozomara and Griffiths-Jones, 2011, 2014; Zhang, 2015; Kozomara et al., 2019). However, only a small fraction of known miRNAs have so far been studied. Overexpressing certain miRNAs or expressing of the resistant version of a target mRNA allows the roles of miRNAs to be investigated by analyzing the resulting phenotypes. The characterization of loss-of-function mutants of *MIR* genes and the strategy of interfering with mature miRNAs have also been successfully employed to identify miRNA functions (Baker et al., 2005; Allen et al., 2007; Franco-Zorrilla et al., 2007; Sieber et al., 2007; Voinnet, 2009; Liu et al., 2010; Todesco et al., 2010; Yan et al., 2012).

Thus far, the general and specific functions of miRNAs have mainly been studied in *Arabidopsis* (*Arabidopsis thaliana*) and *Populus* (poplar; *Populus trichocarpa*). The miRNAs from these species share similar and yet different functions. For example, miR397 is a conserved miRNA in flowering plants (Kozomara and Griffiths-Jones, 2011). In the woody model plant *P. trichocarpa*, miR397 is the main regulator of lignin biosynthesis (Lu et al., 2013). In addition, miR397b affects lignin biosynthesis by regulating its target gene *LACCASE4* (*LAC4*) in *Arabidopsis* (Wang C. Y. et al., 2014). These findings indicate that miR397 has a conserved role in the regulatory network of lignin biosynthesis in herbaceous and woody plants. However, upregulating miR397 expression increased grain size and panicle branching in rice (*Oryza sativa*) by regulating the expression of its target gene *LAC* and thereby significantly improving grain yield (Zhang Y. C. et al., 2013), indicating that conserved miRNAs might exhibit different functions across species. Therefore, identifying and characterizing miRNAs in woody plants would greatly facilitate further research on the evolution and functions of miRNAs.

According to the miRNA database (^1^, Release 22.1; Kozomara et al., 2019), among the 10,414 mature miRNAs identified in 82 plant species, 2,656 were from 26 tree species. Information about plant miRNAs is also hosted in other databases, such as PmiREN (^2^, Guo et al., 2020), a comprehensive functional plant miRNA database that shares 44 species with miRBase. PmiREN contains 20,388 miRNA loci (MIR) belonging to 5,757 families in 88 species ranging phylogenetically from chlorophytes to angiosperms. (The data of the two databases are listed in Table 1).

**TABLE 1:** MiRNAs of trees retrieved from the miRBase and PmiREN.

Species	Abbreviations	Number (miRBase/PmiREN)	References
*Acacia auriculiformis*	*Aau*	7	Wong et al., 2011
*Acacia mangium*	*Amg*	3	Wong et al., 2011
*Amborella trichopoda*	*Atr*	129/186	Amborella Genome Project et al., 2013; Hajieghrari et al., 2015
*Avicennia marina*	*Ama*	3	Khraiwesh et al., 2013
*Bruguiera cylindrica*	*Dcy*	4	Lee et al., 2011
*Bruguiera gymnorhiza*	*Bgy*	4	Lee et al., 2011
*Carica papaya*	*Cpa*	81/97	Ming et al., 2008; Aryal et al., 2012
*Citrus clementina*	*Ccl*	5/137	Song et al., 2009
*Citrus reticulata*	*Crt*	4	Song et al., 2009
*Citrus sinensis*	*Csi*	246/290	Liu et al., 2014; Lu et al., 2014; Taylor et al., 2017
*Citrus trifoliata*	*Ctr*	6	Song et al., 2009
*Cunninghamia lanceolata*	*Cln*	4	Wan et al., 2012
*Elaeis guineensis*	*Egu*	6	Mehrpooyan et al., 2012
*Eugenia uniflora*	*Eun*	74	Guzman et al., 2012
*Gossypium arboreum*	*Gar*	1/181	Farooq et al., 2017
*Hevea brasiliensis*	*Hbr*	30/213	Gébelin et al., 2013
*Malus domestica*	*Mdm*	322	Xia et al., 2012; Yao et al., 2015; An et al., 2018; Feng et al., 2017
*Manihot esculenta*	*Mes*	175/154	Patanun et al., 2013; Ballén-Taborda et al., 2013; Chen X. et al., 2015
*Picea abies*	*Pab*	600/545	Xia et al., 2015
*Pinus densata*	*Pde*	29	Wan et al., 2012
*Pinus taeda*	*Pta*	36	Lu et al., 2007
*Populus euphratica*	*Peu*	4/174	Li B. et al., 2009; Li et al., 2011; Li B. et al., 2013; Si et al., 2014; Lu et al., 2017
*Populus trichocarpa*	*Ptc*	401/265	Lu et al., 2005; Tuskan et al., 2006; Zhao J. P. et al., 2012; Baucher et al., 2013; Lu et al., 2013; Peng et al., 2013
*Prunus persica*	*Ppe*	214/293	Zhu et al., 2012; Eldem et al., 2012; Zhang C. et al., 2016
*Theobroma cacao*	*Tcc*	82	Argout et al., 2011
*Vitis vinifera*	*Vvi*	186/289	Jaillon et al., 2007; Sun et al., 2015; Pantaleo et al., 2016; Snyman et al., 2017;
*Actinidia chinensis*	*Ach*	182	Huang et al., 2013
*Citrus maxima*	*Cit*	208	Wang X. et al., 2017
*Fraxinus excelsior*	*Fex*	203	Sollars et al., 2017
*Ginkgo biloba*	*Gbi*	280	Guan et al., 2016
*Gossypium barbadense*	*Gba*	330	Ibrahim et al., 2006
*Hibiscus syriacus*	*Hys*	679	Kim et al., 2017
*Jatropha curcas*	*Jcu*	94	Vanessa et al., 2014
*Olea europaea*	*Oeu*	266	Yanik et al., 2013
*Persea americana*	*Pam*	92	Rendón-Anaya et al., 2019
*Quercus robur*	*Qro*	134	Ramos et al., 2018
*Phoenix dactylifera*	*Pda*	124	Yaish et al., 2015
*Punica granatum*	*Pgr*	33	Saminathan et al., 2016

Based on the identified miRNA families in plants and their sequence homology, cross-species comparisons have been performed to determine the conservation of certain miRNA families in different plant lineages (Jones-Rhoades, 2012). In this review, we compare the miRNAs of 25 tree species with those of the model plants *A. thaliana*, *O. sativa*, and *P. trichocarpa*. MiRNAs that are found in model plants and at least three woody tree species with high sequence homology are defined as conservative miRNAs. Based on these criteria, 22 highly conserved miRNAs families have been identified in plants, including miR156, miR159/miR319, miR160, miR162, miR164, miR166, miR167, miR168, miR169, miR170/171, miR172, miR319, miR390, miR393, miR394, miR395, miR396, miR397, miR398, miR399, miR408, and miR827 families. These findings are consistent with the results of a previous analysis of flowering plants (Cuperus et al., 2011). Conserved miRNAs families also include miR403, miR828, miR2111, miR477, miR482, and miR3627 (Solofoharivelo et al., 2015). Here, we focus on the potential functions of miRNAs in growth, development, and responses to abiotic and biotic stresses in woody plants.

## Growth and Development

### Wood Formation

Lignin, an important component of the secondary cell wall, forms support tissues in vascular plants. In Chinese white poplar (*Populus tomentosa*), 36 miRNAs were shown to be involved in wood formation by targeting lignin biosynthesis genes (Lu et al., 2005; Quan et al., 2016, 2019). Overexpression of miR6443 in *P. tomentosa* specifically downregulated the expression of the miR6443 target gene, *FERULATE 5-YDROXYLASE 2 (F5H2*), resulting in a significant decrease in S lignin content (Fan et al., 2019). During vascular development in *P. tomentosa*, miR319a and *PtoTCP20* are differentially expressed; overexpression of miR319a in seedlings caused a delay in secondary growth and a decreased xylem yield. A dual-luciferase assay confirmed the targeted cleavage of *PtoTCP20* by miR319a. Yeast two-hybrid and yeast one-hybrid screens demonstrated that PtoTCP20 interacts with PtoWOX4a and activates the expression of *PtoWND6A/B*, thereby promoting the differentiation of cambium into secondary xylem cells and regulating the secondary vascular tissue development (Hou et al., 2020).

In hybrid aspen (*Populus tremula* × *Populus alba*), *Pta*-miR166 targets the Class III HD-Zip transcription factor-encoding gene *Pta-HB1* (the ortholog of *Arabidopsis REVOLUTA*). The expression patterns of *Pta-HB1* are associated with the wood formation in aspen and vary upon seasonal changes (Ko et al., 2006). Moreover, knocking out the Class III HD ZIP transcription factor gene *POPCORONA* led to aberrant lignification in pith cells; conversely, expression of the miR165/166-resistant gene *POPCORONA* delayed the lignification of xylem and phloem fibers during secondary growth (Du et al., 2011). HD-Zip III subfamily genes, the targets of miR165/166, were also highly expressed in the cambium and xylem of plum blossom (*Prunus mume*) and *Acacia mangium*, indicating that miR165/166 is indispensable for vascular differentiation (Ong and Wickneswari, 2012; Li et al., 2019). Interestingly, miR166 was not detected in the xylem of apple (*Malus domestica* “Royal Gala”) (Varkonyi-Gasic et al., 2010).

MiR397a negatively regulates the lignin-biosynthesis-related *LAC* genes in poplar: *P. tomentosa* overexpressing *Ptr-MIR397a* showed decreased expression levels of 17 *Ptr-LAC* genes as well as reduced lignin content (Lu et al., 2013; Chen J. et al., 2015). Similarly, in alpine ash (*Eucalyptus grandis*), 17 *LAC* genes were predicted to be targets of miR397, of which 9 were mainly expressed in vascular tissues involved in the synthesis of xylem lignin (Arcuri et al., 2020). Additionally, degradation sequencing revealed that MYB, the target of miR159, is involved in the wood formation of *E. grandis* (Pappas et al., 2015). In cork oak (*Quercus variabilis*), miR167, miR165/166, miR396, and miR159 are highly expressed in phellogen, pointing to roles in cork formation (Chaves et al., 2014). A recent study comparing the miRNA transcriptomes of different tissues of *P. alba* × *P. glandulosa* and *Larix kaempferi* showed that miRNAs have different roles in regulating wood formation in these two tree species, providing a basis for understanding the wood formation mechanisms of gymnosperms and angiosperms (Li et al., 2020).

The functions of miRNAs are affected by allelic variation; one single-nucleotide polymorphism (SNP) in the pre-miRNA region of *Pto*-MIR160a altered the number of loops in its secondary structure and was significantly associated with tree growth and wood properties (Tian et al., 2016). In addition, variation of an SNP at the binding site of a target gene affected its binding affinity to miRNA (Chen J. et al., 2016). Target gene prediction and transcriptome sequencing suggested that miR257 and its 12 potential target genes control wood formation and tree growth. SNP association analysis to explore the functions of *Pto*-miR257 and its 12 target genes revealed a significant epistatic interaction between *Pto*-miR257 and these genes, expanding our understanding of gene regulation and the mechanisms underlying how miRNA-target interactions affect phenotypic variation (Chen B. et al., 2016).

Single-nucleotide polymorphisms in miRNA biogenesis genes (miRBGs) associated with lignin can alter the expression correlation of miRBG with miRNA and miRNA with target genes, indicating that miRBG mutations regulate the expression of downstream target genes by affecting miRNA expression abundance. Thus, a miRBG-miRNA-target epistasis interaction network that affects forest growth and wood quality variation was constructed, providing a new theory and strategy for the genetic improvement of forests (Chen B. et al., 2018). The allelic variation and interaction between the *Pto*-miR319 family and its 12 target genes related to wood formation were recently analyzed based on their association, representing a step forward in the use of association genetics to explain the multigene interactions of trees (Si et al., 2020).

### Flowering Time and Flower Development

miRNAs such as miR156 and miR172 play important roles in the reproductive development of trees, which are similar to their functions in the model plant *Arabidopsis*. In small Philippine acacia (*Acacia confus*a), Cole’s wattle (*Acacia colei*), blue gum (*Eucalyptus globulus*), ivy (*Hedera helix*), German oak (*Quercus acutissima*), and Canadian poplar (*Populus* × *Canadensis*), miR156 expression levels remain high at the seedlings stage and decrease during the juvenile-to-adult transition. However, the expression pattern of miR172 is different (Wang et al., 2011). An integrated analysis of mRNA and miRNA found that the MsSVP/miR172/mssap2-like module controls the expression of *MsLFY* by responding to gibberellin signals and stimulates the transition from vegetative growth to reproductive growth in *Magnolia* × *soulangeana* (Sun et al., 2020). Furthermore, overexpression of miR156 in transgenic Canadian poplar plants reduced the expression of miR156-targeted SPL genes and miR172, leading to the prolonged juvenile phase (Wang et al., 2011). Overexpression of miR172a caused early flowering, aberrant flowers formation, and abnormal leaf development in physic nut (*Jatropha curcas*) (Tang et al., 2018). Similarly, the expression level of miR156 was high, whereas the expression levels of miR172 was low, in the leaves of Chinese crabapple (*Malus hupehensis*) at the juvenile phase (Xing et al., 2014). The finding that miR156 and miR172 have similar expression patterns in other woody plants, such as trifoliate orange (*Poncirus trifoliata*) (Zhu and Helliwell, 2011; Zhang X. N. et al., 2014), kiwifruit (*Actinidia deliciosa*) (Erika et al., 2012), Chinese plum (*Prunus mume*) (Wang T. et al., 2014), apple (*M. domestica*) varieties “Golden Delicious” (Guo et al., 2017), Fuji, Fuji/M9, and M9 (An et al., 2018), and Rhododendrons (*Rhododendron arboreum*), implies that these two miRNAs have conserved functions in controlling the juvenile-to-adult transition (Choudhary et al., 2018).

In Chinese hickory (*Carya cathayensis*), miR156, miR160, miR167, miR172, and miR394 were upregulated during flower development (Wang et al., 2012). Likewise, miR156, miR166, miR167, miR169, and miR171 together with their target genes were differentially expressed, providing further support for critical roles of miRNAs in male and female flower development in *C. cathayensis* (Sun et al., 2017). The target *ARF4* genes of miR167, miR529, and miR2950 affect flower development in Chinese jujube (*Ziziphus jujuba*) infected by phytoplasma, causing floral organs to develop into leaf-like structures (Ma et al., 2020). Nine miRNAs (miR157a/b, miR161, miR164b, miR166a/b, miR172, miR399, and miR776) are expressed in mature or germinated pollen of loblolly pine (*pinus taeda*), pointing to their potential functions in pollen germination and pollen tube growth (Quinn et al., 2015). In addition, miR157, miR160, miR167, and miR171 might control the induction of female flowers formation in walnut (*Juglans regia*), since they were mainly detected in female flowers (Zhou et al., 2018). The target gene of miR156 was mapped to the gender locus in dustpan willow (*Salix suchowensis*), pointing to the function of miR156 in controlling flower gender (Wei et al., 2017). Furthermore, the methylation of miR172b might play an important role in flower gender determination, as miR172b is hypermethylated in female *P. tomentosa* flowers (Song et al., 2013, 2015).

It is likely that miR156, miR164, miR166, and miR5139 control rose petal development by regulating the ethylene signaling (Kim et al., 2012; Pei et al., 2013). An analysis of differentially expressed miRNAs in simple flowers (wild-type) and double flowers (mutant) of shiny-leaved yellowhorn (*Xanthoceras sorbifolia*) suggested that miR159, miR164, miR166, and miR319 are involved in floral organ development (Ao et al., 2012). In *M. domestica*, miR159 might control male organ and embryo development by targeting *MYB* genes (Xia et al., 2012). Moreover, expressing the precursor of miR396c of *P. trichocarpa* in tobacco altered the identity of stamens and carpels, suggesting that miR396 is essential for floral organ specification (Baucher et al., 2013).

### Seed Development

MiRNAs play important roles in seed development, dormancy, and germination. MiR156b, miR414, miR2673b, and miR7826 control seed development by regulating the expression of genes required for fatty acid biosynthesis and lipid metabolism in tree peony (*Paeonia suffruticosa*) (Yin et al., 2018). The seed oil of *J. curcas* is a promising substitute for petro-diesel. A variety of miRNAs, including miR156, miR157, miR159, miR166, miR167, miR168, and miR396, target genes associated with cellular structure, nuclear function, translation, transport, hormone synthesis, and lipid metabolism during seed development in *J. curcas* (Galli et al., 2014). Similarly, deep sequencing of the woody oil seed plant of *Pongamia* (*Pongamia pinnata*) at three stages of development (embryogenesis phase, seed-filling phase, and desiccation phase) revealed that miR482a-3p regulates fatty acid biosynthesis during seed development (Jin et al., 2019). In coconut (*Cocos nucifera*), miR166a, miR397a, and miR398a are highly expressed in the immature endosperm (Li D. et al., 2009). Additionally, during somatic embryogenesis in longan (*Dimocarpus longan*), many miRNAs were preferentially expressed at different stages: miR156 and miR166c^∗^ are expressed in the early embryonic cultures; miR160, miR159, miR390, and miR398 are expressed during the heart-shaped and torpedo-shaped stages; miR167, miR168a^∗^, miR397, miR398, miR808, and miR5077 are expressed during the cotyledon stage; while miR167, miR808, and miR5077 are expressed during the mature embryo stage (Lin and Lai, 2013). In loblolly pine (*P. taeda*), miR166 is mainly expressed in zygotic embryos and female gametophytes. However, miR167 was only detected in zygotic embryos, representing the first report of miRNAs in gametophytic seed tissues (Oh et al., 2008). In Japanese larch (*Larix kaempferi*), miR-13, miR1313b, miR-14, miR319a, miR894g, miR159c, miR-12, miR397d, and miR162a are upregulated in dormant embryos, whereas miR168b, miR169b, miR4414b, and miR529a are upregulated in germinated embryos (Zhang J. et al., 2013). Similarly, overexpression of miR166 suppressed the formation of cotyledons during somatic embryogenesis, leading to abnormal development of the apical embryo (Li et al., 2016). MiR159 maintains somatic embryo maturation by negatively regulating its target *LaMYB33* (Li W. F. et al., 2013). Finally, miR397 is involved in the process of converting dormant embryos into germinating embryos by regulating the thickness of cell walls. During this process, abscisic acid alters composition of miRNAs by regulating the expression levels of miRNA biogenesis genes (Zhang J. et al., 2013).

### Fruit Development

MiRNAs, particularly miR156, miR160, miR167, miR172, miR390, miR393, miR397, miR828, and miR858, are important for fruit development (Damodharan et al., 2016; Chen C. et al., 2018). In *M. domestica*, the silencing of miR172 *via* a transposon insertion led to an increase in fruit size; conversely, overexpressing miR172 significantly reduced fruit size and altered flower structure (Yao et al., 2015). MiR408 regulates the biomass of *Arabidopsis* and rice: overexpressing *MIR408* in these plant significantly enhanced photosynthesis by improving light utilization efficiency and carbon dioxide fixation capacity, thereby increasing biomass and seed yield (Pan et al., 2018; Song et al., 2018). In rice, miR408 can increase yield by regulating its target *UCL8*, encoding a member of the phytocyanin family, to increase the number of panicles and grains (Zhang et al., 2017). In addition, in *P. trichocarpa*, *ptr*-miR408 effectively targets plastocyanin-like protein genes (*PCLs*) (Lu et al., 2005, 2008). Although miR408 regulates different target genes in herbaceous and woody plants, all of these targets encode copper-containing proteins that are essential for photosynthetic electron transport. Therefore, miR408 is thought to plays an important role in the photosynthesis and fruit yield of woody plants.

In tea-oil camellia (*Camellia oleifera*), miR156, miR390, and miR395 are involved in regulating carbohydrate accumulation during fruit growth and development, and miR477 is a key factor in fatty acid synthesis (Liu et al., 2019). In litchi (*Litchi chinensis*), the miR156-targeted gene *Lc-SPL1*/*2* encodes a transcription factor that interacts with *Lc-MYB1* to control fruit coloration by promoting anthocyanin biosynthesis (Liu et al., 2016). Similarly, miR156, miR159, miR828, miR858, and miR5072 regulate the accumulation of anthocyanin in apple peel (Xia et al., 2012; Qu et al., 2016). Furthermore, restricting root cultivation in grapevine (*Vitis Vinifera*) inhibits the expression of miR828, which in turn enhances anthocyanin metabolism by increasing the expression of its potential target *MYB* genes (Chen et al., 2019). In barbary wolfberry (*Lycium barbarum*), miR156/157 and their target genes *Lb-CNR* and *Lb-WRKY8*, together with miR171 and its target gene *Lb-GRAS*, are thought to be involved in controlling the fruit ripening process. Finally, miR156 negatively regulates the expression of fructokinase and 1-deoxy-D-xylulose-5-phosphatesynthase genes in *L. barbarum*, and miR164 targets β-fructofuranosidase genes to control the quality and nutritional value of fruits (Zeng et al., 2015).

The secondary thickening of the parenchyma cell wall and the deposition of lignin result in the formation of stone cells during fruit development (Cai et al., 2010; Yan et al., 2014). Profiling of miRNAs expression in pear fruits at different developmental stages found that many miRNAs play essential roles in fruit development by regulating the expression of genes involved in lignin synthesis, glycometabolism, acid metabolism, and hormone signaling (Wu et al., 2014). Specifically, miR3711, miR419, and miR5260 downregulate the hydroxycinnamoyl transferase (*HCT*) gene, which promotes lignin formation (Wu et al., 2014). Examination of miRNAs in pear fruits with different amounts of stone cells revealed that miR1809 and miR144-3p negatively regulate genes encoding laccases (*LAC)*, which are key lignin biosynthesis enzymes (Cheng et al., 2017). In addition, miR397 targets *LAC* genes to control fruit cell lignification in Chinese pear (*Pyrus bretschneideri*) (Xue et al., 2018). Finally, many *ARF* genes, which are potential targets of miR160 and miR167, act as repressors of fruit abscission in *L. chinensis* (Zhang et al., 2019).

**FIGURE 1:**
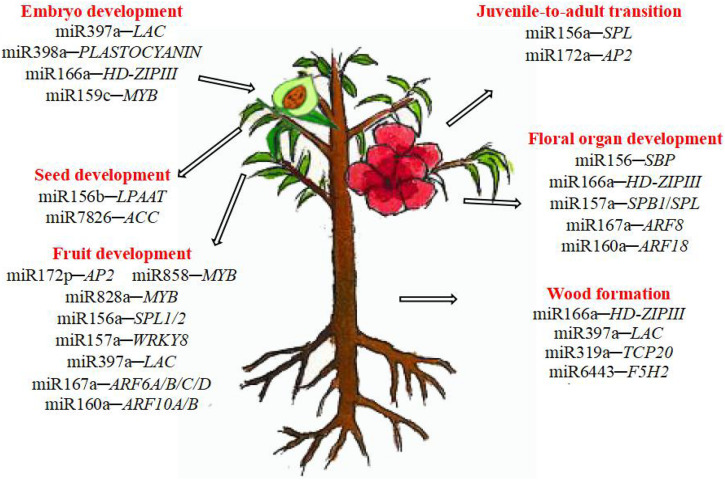
The role of miRNA in the growth and development of woody plants.

MiRNAs are involved in various stages of woody plant growth (Figure 1). Studies using various approaches suggested that miRNAs largely target genes encoding transcription factors that regulate plant growth, development, and hormone signaling. The same miRNAs are sometimes involved in different stages of woody plant only limited studied of this topic development, and different miRNAs sometimes regulate the same type of targets, highlighting the complexity of post-transcriptional regulation *via* miRNAs. MiRNAs involved in growth and development of trees are summarized in Table 2.

**TABLE 2:** MiRNAs are involved in the growth and development of trees.

Species	miRNA name	Potential target gene(s)	Sequence (5′-3′)	Potential function	References
*Populus canescens* (*Populus tremula* × *Populus alba*)	miR166a	*HD-ZIPIII* (5′ RACE)	ucggaccaggcuucauucccc	Wood formation	Ko et al., 2006; Du et al., 2011
	miR397a	*LAC* (Overexpression of transgenic lines)	ucauugagugcagcguugaug		Lu et al., 2013; Chen J. et al., 2015
*Populus tomentosa*	miR319a	*TCP20 [*Luciferase reporter gene (LUC) assay*]*	uuggacugaagggagcuccc		Hou et al., 2020
	miR6443	*F5H2* (Degradome)	guaugaucauagaugauggag		Fan et al., 2019
*Eucalyptus grandis*	miR159a	*MYB* (Degradome)	uuuggauuuaagggagcucua		Pappas et al., 2015
*Jatropha curcas*	miR156a miR172a		ugacagaagagagugagcac agaaucuugaugaugcugcau	Juvenile-to-adult transition	Tang et al., 2018
*Malus hupehensis*					Xing et al., 2014
*Malus domestica*					Guo et al., 2017; An et al., 2018
*Rhododendron*		*SPL* (Degradome sequencing) *AP2* (Degradome)			Choudhary et al., 2018
*Poncirus trifoliata*					Zhu and Helliwell, 2011; Zhang X. N. et al., 2014
*Prunus mume*					Wang T. et al., 2014
*Actinidia deliciosa*					Erika et al., 2012
*Carya cathayensis*	miR156	*SBP* (psRobot)	ugacagaagagagugagcac	Floral organ specification and development	Sun et al., 2017
	miR166a	*HD-ZIPIII* (psRobot)	ggaauguugucuggcucgagg		
*Juglans regia*	miR157a-5p	*SPB1*/*SPL* (psRobot)	uugacagaagauagagagcac		Zhou et al., 2018
	miR167a-5p	*ARF8* (psRobot)	ugaagcugccagcaugaucua		
	miR160a-5p	*ARF18* (psRobot)	ugccuggcgcccuguaugcca		
*Populus tomentosa*	miR172b	*AP2* (5′ RACE)	agaaucuugaugaugcugcau		Song et al., 2013; Song et al., 2015
*Populus trichocarpa*	miR396c	*GRF* (5′ RACE)	uuccacagcuuucuugaacuu		Baucher et al., 2013
*Malus domestica*	miR159a	*MYB* (5′ RACE)	agaaucuugaugaugcugca	Male organ and embryo development	Xia et al., 2012
*Paeonia suffruticosa*	miR156b	*LPAAT* (TargetFinder)	ugacagaagagagugagcac	Seed fatty acid metabolism	Yin et al., 2018
	miR7826	*ACC* (TargetFinder)	caaaauuugaaacuuggug		
*Cocos nucifera*	miR397a	*LAC* (miRU web server)	ucauugagugcagcguugaug	Embryo development	Li D. et al., 2009 Xia W. et al., 2014
	miR398a	*PLASTOCYANIN* (miRU web server)	uguguucucaggucaccccuu		
*Larix leptolepis*	miR166a	*HD-ZIPIII* (Transgenic)	gaccaggcuucauuccccaa		Li et al., 2016; Li W. F. et al., 2013
	miR159c	*MYB* (5′ RACE)	cuuggauugaagggagcucca		
*Malus domestica*	miR172p	*AP2* (Transgenic)	agaaucuugaugaugcugcau	Fruit size	Yao et al., 2015
	miR858	*MYB* (Degradome)	uucguugucuguucgaccuga	Anthocyanin accumulation	Xia et al., 2012; Qu et al., 2016
	miR828a	*MYB* (Degradome)	ucuugcucaaaugaguauucca		
*Litchi chinensi*	miR156a	*SPL1/2* (Degradome)	agcgcguugacagaagagg		Liu et al., 2016
*Vitis Vinifera*	miR828a	*MYB113* (psRNATarget)	ucuugcucaaaugaguauucca		Chen et al., 2019
*Lycium barbarum*	miR171a	*GRAS*(psRobot)	ugauugagccgcgccaauauc	Fruit ripening	Zeng et al., 2015
	miR156a	*CNR* (psRobot)	ugacagaagagagugagcac		
	miR157a	*WRKY8* (psRobot)	uugacagaagauagagagcac		
*Pyrus bretschneideri*	miR397a	*LAC* (5′ RACE)	ucauugagugcagcguugaug	Fruit cell lignification	Xue et al., 2018
*Litchi chinensi*	miR167a	*ARF6A/B/C/D* (psRNATarget)	ugaagcugccagcaugaucuga	Fruit abscission	Zhang et al., 2019
	miR160a	*ARF10A/B* (psRNATarget)	gaucauguucgcaguuucacc		

## Abiotic Stress

### Heat Stress

Heat stress affects the growth and development of trees. Both conserved and non-conserved heat-responsive miRNAs have been identified in various plant species. However, only limited studies of this topic have been conducted in just a few woody plants. In *P. trichocarpa*, miR156, miR159, miR164, miR171, and miR408 are responsive to stress in a tissue-specific manner (Lu et al., 2005). In addition, 52 miRNAs in *P. tomentosa* were found to respond to heat stress, most of which are downregulated (Chen et al., 2012a). In *Betula luminifera*, downregulation of the miR162-targeted gene *DCL1* and the miR403-targeted gene *AGO2* might modulate miRNA biosynthesis, thereby altering the abundance of miRNAs and their responses to heat stress (Pan et al., 2017). Furthermore, in cassava (*Manihot esculenta*), miR156a, miR159, miR160, miR397, and miR408 are downregulated by heat stress, indicating that they play important roles in heat stress responses by negatively regulating their target genes *MYB* and *LAC* (Ballén-Taborda et al., 2013).

### Cold Stress

Cold stress negatively affects the growth and development of trees and restricts their spatial distribution. In *P*. *trichocarpa*, 156 genes were identified as potential targets of 14 cold-responsive miRNAs (Lu et al., 2008). The role of miR475b in the freezing response has been reported in sweet poplar (*Populus suaveolens*) (Niu et al., 2016). Cold stress induces the expression of similar miRNAs between *P. suaveolens* and *P. trichocarpa* with few exceptions, such as miR168a, miR168b, and miR475, suggesting that miRNAs play partially conserved roles in the responses of these trees to cold stress (Sun, 2011). MiR319a-c and miR395b-k are downregulated by cold stress, in *P. tomentosa*, resulting in enhanced resistance to this stress by regulating their targets *MYBs* and *APS* (Chen et al., 2012c). In addition, 21 upregulated and 43 downregulated miRNAs and 46 upregulated and 45 downregulated miRNAs were predicted to respond to cold stress in the tea plant (*Camellia sinensis*) varieties in “Yingshuang” and “Baiye 1,” respectively (Zhang Y. et al., 2014). In almond (*Prunus dulcis*), cold stress increases the expression of miR159, miR164a-c, miR171a/c/g/f, miR394a/b, miR482f, and miR7122a-5p, but decreases the expression of miR156a/b, miR162, miR166a-e, miR396b, miR398a-3p, miR6262, and miR8123-5p (Karimi et al., 2016). Furthermore, miR7122-3p might target the cold-tolerance gene *Pd-HOS1* (Alisoltani et al., 2015). MiR169 is expressed at low levels in a cold-tolerant clone of rubber tree (*Hevea brasiliensis*), suggesting that miR169 might affect the expression of its target gene *NF-YA* to enhance cold resistance (Kuruvilla et al., 2017). In grapevine (*V. vinifera*), cold-induced miRNAs might negatively regulate the expression of various transcriptional factor genes, including *AP2*, *SBP*, *MYB*, *bHLH*, *GRAS*, and *bZIP* (Sun et al., 2015). Moreover, 36 conserved and 5 novel miRNAs are either up or downregulated in response to cold stress in trifoliate orange (*P. trifoliata*) (Zhang X. N. et al., 2014). Further studies showed that miR396b enhances cold tolerance in *P. trifoliata* by negatively regulating the expression of the 1-aminocyclopropane-1-carboxylic acid oxidase (*ACO*) gene, which consequently affects ethylene and polyamine synthesis (Zhang X.N. et al., 2016). In *M. esculenta*, miRNAs participate in the response and acclimation to low temperatures by regulating stress-related pathways, such as auxin signaling (Xia J. et al., 2014). By integrating miRNA and transcriptome analysis of lesser black poplar (*Populus simonii* × *P. nigra*), miR319, miR159, miR167, miR395, miR390, and miR172 were found to be differentially expressed under cold treatment and to negatively regulate their target genes, including *ARF*, *MYB*, *SBP*, *LHW*, and *bZIP*. These target genes played important roles in cold resistance (Zhou et al., 2019).

### Drought Stress

Drought stress can severely affect the growth and productivity of trees. Many miRNAs are involved in regulating the drought response in trees. In *P. trichocarpa*, miR164, miR394, miR408, miR473, miR123, miR1444a, miR93a, miR93b, and their potential target genes are differentially expressed under drought stress, pointing to their essential roles in the drought response (Lu et al., 2008; Kreuzwieser et al., 2009; Li B. et al., 2009; Wilkins et al., 2009; David et al., 2010; Peng et al., 2013). In *V. vinifera*, various miRNAs, such as miR166, miR319, miR396, miR3631, mi3633, and miR3639, are downregulated in response to severe water stress (Pantaleo et al., 2016). In addition, the accumulation of miRNAs including miR156, miR159, miR164, miR166, miR393, miR394, miR396, miR3624, and miR3639 in grapevine varies under drought stress depending on the grape variety and rootstocks (Pagliarani et al., 2017). Moreover, in the stems and leaves of Mongolian *Ammopiptanthus* (*Ammopiptanthus mongolicus*), miR2118 and miR858 upregulate the expression of the *OZF1* and various *MYB* genes that control flavonol production under drought stress (Gao et al., 2016). In *H. brasiliensis*, miR168 and miR160 are associated with drought tolerance (Kuruvilla et al., 2019). In mulberry (*Morus alba*), miR156, miR172, and miR396 as well as their target genes might be involved in the response to drought stress (Li et al., 2017). Additionally, in peach (*Prunus persica*), the expression levels of 262 miRNAs in leaves and 368 miRNAs in roots were significantly altered upon drought stress (Eldem et al., 2012). In citrus (*Citrus junos* Siebold cv. “Ziyang”), 19 known miRNAs and 15 novel miRNAs were shown to respond to dehydration treatment. Among these, 16 miRNAs were downregulated whereas 18 miRNAs were upregulated under these conditions (Xie et al., 2017).

In *P. tomentosa*, several miRNAs, such as pto-miR394, pto-miR160f, pto-miR472, and pto-miR1448, and their target genes showed opposite expression patterns under drought and flooding stress; their target genes encode transcription factors and signal transduction elements that participate in stress responses. These findings further emphasize the importance of miRNAs in plants under water stress (Ren et al., 2012). A recent study showed that Pu-miRNA172d reduces stomatal density, net photosynthetic rate, and transpiration by inhibiting the expression of its target gene *PuGTL1* in Cathay poplar (*Populus ussuriensis*) (Liu et al., 2020). Similar findings have been reported in princess tree (*Paulownia tomentosa*) and caragana (*Caragana korshinskii*), supporting the notion that many miRNAs play important roles in plant responses to drought stress (Deng et al., 2017; Ning et al., 2017).

### Salt Stress

The expression levels of many miRNAs vary in responsive to salt stress (Zhang Q. et al., 2013, Zhang, 2015; Sun et al., 2019). In *P. tomentosa*, salt stress alters the expression of 21 conserved miRNAs (19 downregulated and 2 upregulated) and 7 non-conserved miRNAs (5 downregulated and 2 upregulated) (Ren et al., 2013). In desert poplar (*Populus euphratica*), the expression levels of 15 miRNAs and their putative target genes are regulated by salt stress (Li B. et al., 2013). In addition, the expression level of miR156 significantly increased after salt treatment, and this was followed by the enhanced expression of miR166, miR167, miR169, miR172, miR827, miR2119, and miR5020. By contrast, the expression of miR393, miR645, miR860, and miR1444 decreased following salt treatment (Si et al., 2014). Furthermore, several similar studies have identified many conserved and non-conserved miRNAs that are responsive to salt stress in date palm (*Phoenix dactylifera*) (Yaish et al., 2015), mulberry (*Morus alba*) (Wu et al., 2015), and citrus (*C. junos*) (Xie et al., 2017). The *Salicaceae* family member Cathay poplar (*Populus cathayana*) is salt-sensitive, but the dryland willow (*Salix matsudana*) is highly salt-tolerant. Saline treatment decreased the expression of miR474c and miR398b in *P. cathayana* but increased their expression in *S. matsudana*, suggesting that miRNAs play key roles in the differential responses of different plant ecotypes to salt stress, which occurs *via* different regulatory mechanisms to enhance plant adaption to saline-alkali environments (Zhou et al., 2012). Moreover, in *Caragana intermedia*, miR157a, miR165a, miR172b, and miR396a were upregulated under salt treatment, while their potential target genes were downregulated, suggesting that miRNAs play important post-transcriptional roles in the adaptation of this plant to salt stress (Zhu et al., 2013). In *P. tomentosa* under salt stress, the increased expression of miR390 promotes an increase in the levels of *TAS3*-derived *trans-*acting short-interfering RNAs (tasiARFs) and the degradation of *ARF4* transcripts. The decreased expression of *ARF4* releases auxin signals to promote lateral root growth and to improve salt tolerance (He et al., 2018).

### Other Abiotic Stresses

In sweet orange trees (*Citrus sinensis*), boron deficiency alters the expression of many miRNAs, such as miR159, miR160, miR164, miR393, miR394, miR408, miR472, miR474, miR782 miR821, miR830, miR2118, miR3465, miR5023, and miR5266, pointing to important roles for these miRNAs in boron uptake (Lu et al., 2014). Another study showed that miR397a functions in boron tolerance by targeting *LAC17* and *LAC4*, which control secondary cell walls synthesis (Huang J. H. et al., 2016). Copper is an essential micronutrient for plant photosynthesis. In *Arabidopsis*, when the supply of copper was limited, miR398, miR397, miR408, and miR857 accumulated and downregulated their target genes, encoding a copper/zinc superoxide dismutase, a copper protein phytocyanin, and members of the copper protein family of laccases. This mechanism allows plants to preserve copper for some important functions (Abdel-Ghany and Pilon, 2008). Similar to the pattern in *Arabidopsis*, the target genes of miR397, miR398, and miR408 were also downregulated in *P. trichocarpa* under copper deficiency. In addition, miR1444, a *Populus*-specific miRNA, participates in the copper regulatory network by regulating the expression of its target polyphenol oxidase gene (Lu et al., 2011).

miR167 was significantly upregulated in roots of coffee (*Coffea arabica*) at the beginning of a period of nitrogen starvation. The target gene of miR167 encodes the auxin response factor ARF, which is involved in lateral roots development. The expression of miR159 increased after 10 days of nitrogen starvation; its target MYB and TCP genes play important roles in regulating root activity. These findings indicate that this miRNA responds to nitrogen starvation by regulating different biological pathways at different time points (Zhao M. et al., 2012; dos Santos et al., 2019). Similarly, nitrogen deficiency increases the expression of miR159, miR160, miR166, miR167, miR169, miR171, miR172, miR390, and miR475 but decreases the expression of miR393 and miR395 in *P. tomentosa* (Ren et al., 2015). A recent study showed that low-nitrogen-responsive miRNAs, including miR167 and miR171, also participate in the response to low phosphorus in *P. tomentosa* (Bao et al., 2019).

Low sulfur levels induce the expression of miR395 and repress the expression of the miR395 target gene *ATPS3* in poplar, thus inhibiting sulfur assimilation; high sulfur levels inhibits the expression of miR399, whose target gene *PHO2* enhances sulfur assimilation (Shi et al., 2020). In several herbaceous plants, miR395 and miR399 accumulate in the phloem under sulfur and phosphorus deficiency and are transported from the stem to the root, where they regulate the uptake of sulfur and phosphorus by inhibiting the expression of their target genes *PHO2* and *APS4.* In addition, these miRNAs are transported through the xylem to aboveground tissue, thereby regulating the homeostasis of the plant nutrition system (Pant et al., 2008; Buhtz et al., 2010; Chiou and Lin, 2011; Kehr, 2013). A similar phenomenon has been observed in *B. luminifera*: miR399 and miR395 were significantly upregulated under low -phosphorus conditions. Moreover, *PLDD* was identified as the cleavage target of miR399c (Zhang et al., 2021). These findings indicate that herbaceous species and woody perennials adapt to low-phosphorus environments by regulating different targets.

In sycamores (*Platanus acerifolia*), *pla*-miR482a-3p and *pla*-mir39 were differentially expressed under lead stress. Their targets are *RPM1* and *NBS*, respectively, which encode disease resistance proteins, suggesting that lead stress induces signal transduction pathways related to plant defense against pathogens (Wang et al., 2015). In *P. trichocarpa*, 12 PtrDof genes appear to be targeted by 15 miRNAs, seven of which were upregulated in leaves and roots under osmotic stress at all time points, indicating that PtrDof transcription factors play an important role in the resistance of this tree to osmotic stress (Wang H. et al., 2017).

### Biotic Stress

The fact that diseases affect the expression of many miRNAs and their target genes supports the idea that miRNAs play diverse roles in biotic stress responses. In *P. trichocarpa*, the potential target genes of miR472, miR482, miR1447, and miR1448 are disease-resistance genes (Lu et al., 2008). Twelve miRNAs (miR156, miR159, miR160, miR164, miR166, miR168, miR172, miR319, miR398, miR408, miR1448, and miR1450) were upregulated in poplar after infection with the stem canker pathogen *Botryosphaeria dothidea* (Zhao J. P. et al., 2012). Similar miRNAs that respond to the canker pathogen *Valsa mali* were identified in *P. beijingensis* (*P. cathayana* × *P. nigra var. italica*) (Chen et al., 2012b). Moreover, miR482b plays an important role in the response of *M. domestica* to the infection by *V. mali* by regulating its target genes, encoding CC-NBS-LRR class resistance proteins (Feng et al., 2017). In Mexican lime (*Citrus aurantifolia*), miR157 might be involved in altering tree structure and leaf morphology in response to phytoplasma infection (Ehya et al., 2013).

Following infection of loblolly pine (*P. taeda*) with fusiform rust gall, miRNAs from 10 families (including miR156, miR159, and miR319) were significantly downregulated in the stems. Although the expression of these miRNAs in the roots and upper stems of the infected parts of the tree did not change significantly, the expression of their target genes significantly increased. These findings suggest that the host produced an immune response in the uninfected parts of the tree following pathogen infection that led to the host’s defense response (Lu et al., 2007). In wax gourd poplar (*Populus purdomii*), 7 conserved and 20 novel miRNAs that are responsive to foliar rust fungus infection are predicted to regulate genes encoding disease-resistance proteins, serine/threonine protein kinases, transcription factors, and other related proteins (Chen and Cao, 2015). Furthermore, in *V. vinifera*, phytoplasma infection induced the differential expression of various conserved and novel miRNAs that might target genes involved in morphology, hormone signaling, nutrient homeostasis, and stress (Snyman et al., 2017). The number of differentially expressed miRNAs reflects the degree of symbiosis between fungi and roots (Mewalal et al., 2019). When *P. tomentosa* was inoculated with endophytic *Streptomyces* sp. SSD49, miR160 and miR156 were upregulated, and miR319 was downregulated. Their targets primarily encode auxin response factors, disease-resistant proteins, plant hormone oxidases, and so on, which could promote plant growth and improve disease resistance (Tian et al., 2019). *NBS-LRRs*, the targets of miR472, launch effective immune responses in the fungus *Cytospora chrysosperma* (Su et al., 2018).

Some miRNAs in the phloem function as signaling molecules for long-distance transport (Notaguchi and Okamoto, 2015). For example, after mulberry was infected by a phytoplasma, the expression of mul-miR482a-5p in the phloem sap increased. Although the expression levels of the target genes of this miRNA were not significantly altered in the phloem, these genes were downregulated in roots. Grafting experiment showed that mul-miR482a-5p could be transported from the scion to rootstock. Therefore, mul-miR482a-5p in the phloem sap may regulate its target genes in roots (Gai et al., 2018).

**FIGURE 2:**
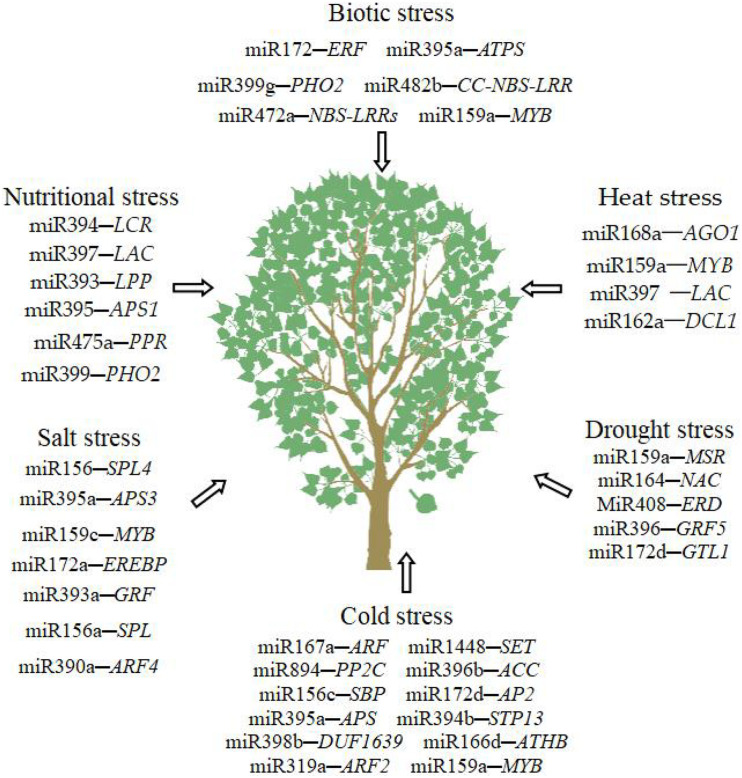
MiRNAs involved in biotic and abiotic stresses in trees.

Many microRNAs are involved in abiotic and biotic stresses (Figure 2). Same miRNAs play vital roles in multiple abiotic and biotic stresses. The miRNAs that are important to the abiotic and biotic stresses of trees are summarized in Table 3.

**TABLE 3:** MiRNAs are important to the biotic and abiotic stresses of trees.

Species	miRNA Name	Potential Target Gene(s)	Sequence (5′-3′)	Potential Function	References
*Populus trichocarpa*	miR168a	*AGO1* (5′ RACE)	ucgcuuggugcaggucgggaa	Heat stress	Lu et al., 2005; Chen et al., 2012a
*Manihot esculenta*	miR159a	*MYB* (PsRNA target)	agcugcugagcuauggauccc		Ballén-Taborda et al., 2013
	miR397	*LAC* (PsRNA target)	uuugagugcagcguugauga		
*Betula luminifera*	miR162a	*DCL1* (Degradome sequencing)	ucgauaaaccucugcauccag		Pan et al., 2017
	miR403a	*DCL1* (Degradome sequencing)	uuagauucacgcacaaacucg		
*Populus trichocarpa*	miR167a	*ARF* (5′ RACE)	ugaagcugccagcaugaau	Cold stress	Lu et al., 2008
	miR1448	*SET* (5′ RACE)	cuuuccaacgccucccauac		
*Poncirus trifoliata*	miR894	*PP2C* (Web MicroRNA Designer)	guuucacgucggguucacca		Zhang X. N. et al., 2014
	miR396b	*ACC* (Transgenic)	uuccacagcuuucuugaa		Zhang X.N. et al., 2016
*Vitis vinifera*	miR156c	*SBP* (5′ RACE)	ugacagaagagagugagcac		Sun et al., 2015
	miR172d	*AP2* (5′ RACE)	ugagaaucuugaugaugcugcau		
	miR395a	*APS* (5′ RACE)	cugaaguguuugggggaacuc		
*Prunus dulcis*	miR394b	*STP13* (5′RACE)	gcggcguuggcauucuguccac		Karimi et al., 2016
	miR398b	*DUF1639* (5′ RACE)	gcggcgcguguucucaggucg		
	miR166d	*ATHB* (5′ RACE)	gcggcgucggaccaggcuuc		
*Populus simonii* × *P. nigra*	miR319a	*ARF2*(psRobot)	uuggacugaagggagcuccc		Zhou et al., 2019
	miR167a	*SBP1*(psRobot)	ugaagcugccagcaugaucua		
	miR159a	*MYB*(psRobot)	uuuggauugaagggagcucua		
	MiR172a	*bZIP44*(psRobot)	agaaucuugaugaugcugcau		
*Populus trichocarpa*	miR159a	*MSR* (Degradome)	uuuggauugaagggagcucua	Drought stress	Li B. et al., 2009; David et al., 2010; Peng et al., 2013
	miR164	*NAC* (Degradome)	uggagaagcagggcacgugca		
	MiR408	*ERD* (Degradome)	cggggaacaggcagagcaugg		
*Vitis vinifera*	miR164	*NAC* (Degradome)	uggagaagcagggcacgugca		Pantaleo et al., 2016;
	miR396	*GRF5* (Degradome)	uuccacagcuuucuugaacua		
*Populus ussuriensis*	miR172d	*GTL1* (5′ RACE)	ggaaucuugaugaugcugcau		Liu et al., 2020
*Populus euphratica*	miR156	*SPL4* (Degradome)	ugacagaagagagugagcac	Salt stress	Li B. et al., 2013
	miR393a-5p	*AFB2* (Degradome)	uccaaagggaucgcauugauc		
	miR395a	*APS3* (Degradome)	cugaaggguuuggaggaacuc		
	miR159c	*MYB* (Degradome)	auuggagugaagggagcucga		Zhou et al., 2012
	miR172a	*EREBP* (Degradome)	agaaucuugaugaugcugcau		
*Caragana intermedia*	miR393a	*GRF* (5′ RACE)	aucaugcuaucccuuuggauu		Zhu et al., 2013
	miR156a	*SPL* (5′ RACE)	ugacagaagagugagcac		
*Populus tomentosa*	miR390a	*ARF4* (transgenic)	aagcucaggagggauagcgcc		He et al., 2018
*Citrus sinensis*	miR394	*LCR* (RNAhybrid)	uuggcauucuguccaccucc	Boron uptake	Lu et al., 2014
	miR397	*LAC* (5′ RACE)	uaguugcgacgugaguuacu		Huang J. H. et al., 2016
*Populus tomentosa*	miR169a	*NF-YA* (psRNATarget)	ucgcuuggugcaggucgggaa	Low nitrogen	Ren et al. 2015;
	miR167	*ARF6/8* (psRNATarget)	ugaagcugccagcaugaucuua		
	miR393	*LPP* (psRNATarget)	uccaaagggaucgcauugauc		
	miR395	*APS1* (psRNATarget)	cugaaguguuugggggaacuc		
	miR475a	*PPR* (psRNATarget)	uuacagugcccauugauuaag		
*Populus tomentosa*	miR399	*PHO2* (Degradome)	ugccaaaggagaauugcccug	Low phosphorus	Bao et al., 2019
*Populus deltoides*	miR395	*ATPS3* (Degradome)	cugaaguguuugggggaacuc	Low sulfate	Shi et al., 2020
	miR399	*PHO2* (Degradome)	ugccaaagaagauuugccccg	Hight sulfate	
*Populus trichocarpa*	miR472b	*Dof30* (5′ RACE)	uuuucccaacuccacccauccc	osmotic stress	Wang H. et al., 2017
*Vitis vinifera*	miR172	*AP2/ERF* (psRNAtarget)	ugaaucuugaugaugcuacau	Phytoplasma infection	Snyman et al., 2017
	miR395a	*ATPS* (psRNAtarget)	cugaaguguuugggggaacuc		
	miR399g	*PHO2* (psRNAtarget)	ugccaaaggagaauugcccug		
*Malus domestica*	miR482b	*CC-NBS-LRR* (Degradome)	ucuuuccuaucccucccauucc	Valsa canker pathogen	Feng et al., 2017
*Populus trichocarpa*	miR472a	*NBS-LRRs* (Degradome)	uuuucccuacuccacccauccc	Fungus *Cytospora chrysosperma*	Su et al., 2018
*Pinus taeda*	miR159a	*MYB* (5′ RACE)	uuggauugaagggagcucca	Fusiform rust gall	Lu et al., 2007

### Conclusion and Future Perspectives

Over the past decade, numerous conserved and species-specific miRNAs have been identified in many woody plants due to the advent of next-generation sequencing technologies. Several studies have revealed that a conserved regulatory mechanism between miRNAs and mRNAs could control various biological processes in different plant tissues. For example, miR166 participates in wood formation (Du et al., 2011), floral organ development (Sun et al., 2017), and embryo development (Li et al., 2016) by regulating the same target HD-Zip III gene. On the other hand, miRNAs can sometimes regulate different biological processes by targeting different genes. *CKB3*, a target of MiR397b, participates in circadian rhythms and affects flowering time in plants (Feng et al., 2020). *LCS9*, the target of miR397, participates in fatty acid metabolism in oil palm (*Elaeis guineensis*) (Zheng et al., 2019). In addition, miR397 is involved in embryo development (Lin and Lai, 2013; Zhang J. et al., 2013) and juice sac granulation (Zhang C. et al., 2016). Furthermore, the targets of miR159, miR828, and miR858 are homologous *MYB* genes with different biological functions. Different miRNAs can bind to the same target but regulate different downstream genes, thus performing different functions. These observations highlight the complexity of miRNA-mediated post-transcriptional regulation. The diversity of woody plants provides a great opportunity to study the evolution of miRNAs.

The bioinformatics tools currently available for miRNA target prediction based on sequence information are PsRobot (Wu et al., 2012), psRNATarget (Dai and Zhao, 2011), and Targetfinder (Kiełbasa et al., 2010). To a certain extent, these approaches allow the target genes of miRNAs to be mined. However, considering that one miRNA can target multiple mRNAs and that one mRNA can also be targeted by multiple miRNAs, the amount of information generated by bioinformatics will be huge, with numerous false-positive candidates. Therefore, it is necessary to identify key genes engaged in a large number of target relationships for experimental verification. At present, some of the functions of miRNAs identified in woody plants can be verified using model plants. However, due to the long generation cycle of perennial woody plants, the establishment of genetic systems for these plants has been restricted. It is difficult to investigate specific aspects of trees in model plants, such as wood formation, seasonal changes, and perennial growth characteristics. Virus-induced gene silencing (VIGS) technology could be utilized for functional verification of miRNAs in woody plants lacking a stable genetic system. CRISPR/Cas9 could be useful for generating a large number of site-directed mutants and to enrich experimental resources. Finally, the combined use of VIGS and CRISPR/Cas9 technology holds great promise for functional analysis of woody plants in the future.

## Author Contributions

YW and LF wrote the manuscript. Both authors contributed to the article and approved the submitted version.

## Conflict of Interest

The authors declare that the research was conducted in the absence of any commercial or financial relationships that could be construed as a potential conflict of interest.

## Publisher’s Note

All claims expressed in this article are solely those of the authors and do not necessarily represent those of their affiliated organizations, or those of the publisher, the editors and the reviewers. Any product that may be evaluated in this article, or claim that may be made by its manufacturer, is not guaranteed or endorsed by the publisher.

## Footnotes

1
http://www.mirbase.org

2
http://www.pmiren.com/
